# COVID-19 case presented initially with keratoconjunctivitis: A case report

**DOI:** 10.1016/j.amsu.2021.102957

**Published:** 2021-10-16

**Authors:** Mohammed Alnajjar, Abdulrahman Al-Mashdali, Nedia Nefatti

**Affiliations:** Division of Internal Medicine, Hamad Medical Corporation, Doha, Qatar

**Keywords:** COVID-19, SARS-CoV-2, Keratoconjunctivitis

## Abstract

**Introduction:**

Ocular complaints are considered non-classical presentations for COVID-19 infection; the initial diagnosis of keratoconjunctivitis is even rarer. Indeed, this puts treating clinicians in danger of getting infected, especially when patients present without the classic respiratory symptoms.

**Case:**

Here we report a case of COVID-19 that was initially presented with keratoconjunctivitis with the appearance of respiratory symptoms four days later. The case showed improvement within four days of successful treatment for both covid pneumonia and ocular disease.

**Discussion:**

Countable cases reported initial ocular symptoms to co-occur with systemic symptoms or even before. Only two cases reported the diagnosis of keratoconjunctivitis in COVID-19. The two cases differ in the proposed mechanism of developing such disorder. One by direct invasion of the virus, the other one by cytokines-induced epithelial injury. Our case did not show positivity for SARS-CoV 2 in the eye secretion, which aligns with the later proposed mechanism of pathology.

**Conclusion:**

It is crucial to report such cases to increase the awareness of atypical presentation for COVID-19 infection. This is too important for two reasons: first, to diagnose the disease itself, and second, to take infection control precautions when treating such cases, with unexpected initial presentation.

## Highlights

• Atypical presentation for COVID-19 infection.
• COVID-19 may initially present with ocular complains.
• Countable cases reported initial ocular symptoms to co-occur with systemic symptoms.
• Infectivity either by direct invasion or cytokines-induced epithelial injury.

## 1 Introduction and importance

Keratoconjunctivitis (KC) is an inflammatory condition that involves both the conjunctiva and the cornea. It usually occurs in association with viral, bacterial, autoimmune, and various allergic conditions. Viral infection is the most common etiology of KC, representing up to 75% of the cases. Adenovirus is the commonest (around 90% of viral KC cases). However, other viruses are also implicated in the pathogenesis of viral KC [1].

In March 2020, the WHO declared a new coronavirus, called COVID-19, a pandemic initially ignited in Wuhan, China. Since then, all reports and expert panels depicted the classical presentation of fever, cough, and generalized fatigue, which progress to pneumonia, to be the predominant clinical picture of the new virus [2].

Angiotensin 2 converting enzyme (ACE-2) receptor, detected in the ocular system with trivial amounts, especially in corneal and conjunctival tissues, is considered the cellular entry receptor for COVID-19 virus [3,4]. A new route of entrance for COVID-19 has been identified by using CD147. This transmembrane glycoprotein was also found in the conjunctiva and cornea, which signifies that SARS-CoV2 could initially affect different eye structures even before involvement of the respiratory tract [5,6]. Since the beginning of the COVID-19 pandemic, only a few COVID-19 cases presented with ocular manifestations have been reported in the literature.

Here, we present a case of COVID-19 that presented initially with isolated ocular symptoms, diagnosed later as keratoconjunctivitis with delayed development of Classic respiratory symptoms. In addition, we reviewed the literature for the previously reported cases of COVID-19 who presented with keratoconjunctivitis and summarized the findings in Table 1.

**Table 1:** Reported cases of COVID-19 presented with ocular manifestations.

	Age	Gender	Ocular symptoms onset	Time-lapse	Initial NP swab PCR-CT value (if available)	Initial conjunctival swab PCR
[14]	65	F	Concurrent with systemic symptoms	N/A	CT 16.1 (done at PSD 1)	CT 21.6 (done at PSD 4)
[15]	27	M	Before systemic symptoms	3 hours	N/A	N/A
[16]	65	M	Before	4 days	N/A (done at PSD 4)	Reported negative (done at PSD 4)
[13]	48	F	Concurrent	Concurrent	N/A	Reported negative
	35	M	Before	Not reported	N/A	N/A
	30	M	Before	Not reported	N/A	N/A
	50	F	Before	One day	N/A	N/A
	57	M	Before	One day	N/A	N/A
	55	M	Before	5 days	N/A	N/A
	40	F	Before	7 days	N/A	N/A
[17]	46	F	Absent systemic signs	N/A	CT 16 (PSD 2)	CT 21 (PSD 2)
	30	M	Absent systemic signs	N/A	CT 32 (PSD 2)	Reported negative (PSD 2)
[11]	29	F	Concurrent	N/A	CT 23 (PSD 8)	CT 37 (PSD 8)
[18]	65	M	Before	3 days	N/A	Reported positive (PSD 13)
	43	F	Before	3 days	N/A	PSD 9, +/-
	28	M	Before	4 days	N/A	Reported positive (PSD 19)
[19]	48	M	Before	2 days	PSD 5, positive	N/A
[20]	41	M	Absent systemic signs	N/A	CT 19 (on Admission)	N/A
	43	M	Absent systemic signs	N/A	CT 22 (on Admission)	N/A
	37	F	Absent systemic signs	N/A	At admission, CT 21	N/A
	65	M	Absent systemic signs	N/A	At admission, CT 17	N/A
	48	M	Absent systemic signs	N/A	At admission, CT 19	N/A
[21]	27	M	Absent systemic signs	N/A	N/A	Reported positive (PSD 2)
[22]	32	M	Absent systemic signs	N/A	PSD 4, positive	Reported negative (PSD 9)

M: Male; F: Female; N/A: Not available; CT: Cycle Threshold; PSD: Post initial symptoms per days.

## 2 Case presentation

A 43-year-old Asian male presented to the emergency department (ED) with left side eye pain and increased lacrimation associated with blurred vision for three days. He also reported a subjective fever since the onset of eye problems, but he denied any respiratory symptoms. He has no significant past medical history, no history of allergy. The patient denied any trauma history and no previous history of a similar incident.

During his stay in the ED, the patient developed one spike of high-grade fever (oral temperature = 38.5C). On examination, the left eye showed profuse clear fluid lacrimation, and there is hyperemia in the inferior nasal quadrant of the left eye. Examination of other systems was unremarkable.

The ophthalmology team was consulted, and comprehensive eyes examination, including slit-lamp and fundoscopy, was performed. Corneal examination showed one central and four paracentral pinpoint scars in the Oculus Sinister (OS). Intraocular pressure was normal in both eyes. Visual acuity was discordant between two eyes with OS 6/18 and normal acuity of Oculus Dextrus (OD).

The patient mentioned that three of his roommates have been complaining of flu-like illness lately. Initial laboratory results were unremarkable, apart from elevated C-reactive protein (50.5 mg/L) and Ferritin (658 μg/L). Given the documented fever and positive history of contact with sick people, a Nasopharyngeal swab for COVID-19 polymerase chain reaction **(**PCR) was sent, which came positive with (CT value of 26.18).

**Fig. 1:**
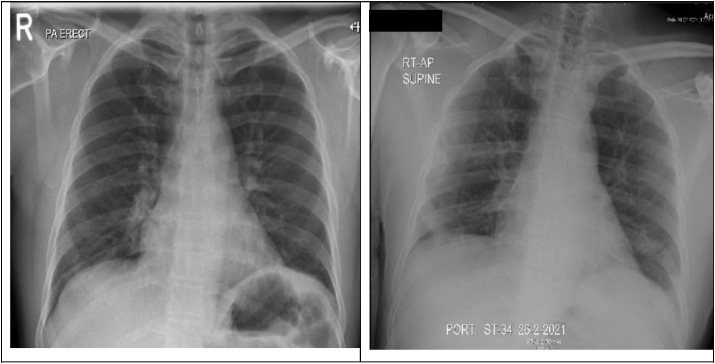
CXR before and after developing respiratory symptoms.

On day two of the hospital stay, the patient started to complain of a productive cough. Chest x-ray was repeated (The first one done in ED was normal) and showed new bilateral lower chest zones infiltrate (Fig. 1). Accordingly, the patient was transferred to a quarantine facility and started on treatment as per national protocol. He was started on an antibiotic, hydroxychloroquine, and lopinavir/ritonavir. Three days post-admission, the patient started to improve in terms of eye pain and secretions.

A swab was taken from the eyes for COVID-19 PCR which came back negative (sample was taken four days after the nasopharyngeal swab).

The patient was discharged from the quarantine facility on day 10 of the hospital stay.

On day 10, after discharge from the quarantine facility, the patient presented to ED due to bilateral orbital pain associated with blurring of vision. He was examined by an ophthalmologist, found to have reduced vision acuity bilateral. Intraocular pressure was high in both eyes; he was diagnosed with closed-angle glaucoma and started on acetazolamide. Currently, the patient is following in the ophthalmology clinic.

## 3 Clinical discussion

Here, we present a COVID-19 case that initially presented with only eyes symptoms. There was around 24 hours lag between the presenting the eye symptoms and the onset of respiratory manifestations. Most previously reported cases with ocular manifestation were diagnosed as conjunctivitis with a delayed ocular manifestation. Although the ocular presentation is rare with this virus, association with keratoconjunctivitis is even rarer.

A recent meta-analysis study showed that out of 8,219 patients with confirmed COVID-19 diagnosis, only 11.03% reported ocular complaints. The most complaint was foreign body sensation which was reported in (16%) of the ocular involved cases, followed by redness (reported in 13.3%), tearing (reported in 12.8%). Most cases are diagnosed with conjunctivitis (88.8%), while 4.4% of cases are diagnosed with either keratitis or keratoconjunctivitis [7].

Seah and colleagues [8] reported that all RT-PCR for tears' of 14 COVID-19 patients were reported negative on repeated occasions. However, all included cases (except one case) did not develop any ocular symptoms during the study timeframe of 14 days. Another prospective study included 30 cases; only one case had an ocular symptom; This case was the only one reported positive for RT-PCR of the eye secretion. The time interval between initial symptoms and positive results was three days [9].

According to Nasiri et al. [3], it was mentioned that the gap between systemic and ocular complaints was 2–21 days (mean 1.5 days), while a shorter gap was reported between ocular and systemic manifestations with a range of 1–3 days (mean 0.04 days). Valente and colleagues showed that the average gap between nasopharyngeal and ocular resolution of the viral load using PCR was four days [10].

To date, two cases in the literature reported keratoconjunctivitis in COVID-19 patients. In one case [11], they did simultaneous RT-PCR of NP and conjunctival swabs in which CT value was discordant between both (23 and 37 cycles, respectively). In our case, there is a four-day gap between taking NP swab and eye swab with PCR results of positive (CT value 26) and negative (i.e., CT > 40 cycle), respectively.

The other case was reported as a relapse case of Keratoconjunctivitis, in which the conjunctival sac swab for COVID-19 RT-PCR was negative. However, the inflammatory markers were high (especially IL-6) in the eye secretions, which could propose another pathophysiology of the disease by local cytokine storm rather than a direct invasion of the virus [12].

There is contradictory data regarding the possibility of viral shedding in eye secretions and whether it is correlated to eye symptoms or not. In one study, 67 cases were enrolled, of which one had ocular symptoms, who tested negative for RT-PCR for COVID-19 taken from eye secretions, while three patients with no eye symptoms tested positive for the virus [13]. On the other hand, in one prospective study, they took a conjunctival swab from 49 COVID-19 patients with no ocular symptoms and sent it for rt-PCR. Four cases tested positive; however, the CT value was high (>30), which means low viral shedding in these patients [14].

Up to our knowledge and after extensive literature review, we summarized the COVID-19 cases that initially presented with ocular manifestation in Table 1.

## 4 Conclusion

In this case, we reported a patient who presented to the hospital mainly due to eye complain, who was initially diagnosed as a case of keratoconjunctivitis. Later during the disease course patient developed respiratory complaints and was diagnosed as a case of COVID-19 infection. This case tells us that the respiratory system is not usually the prime site to be involved and to consider the ocular complaint to be one of the extra-respiratory presenting manifestations of this infection, putting this in the context of pandemic and patient's history of sick contact. This also puts a significant burden on the first responding physician when examining patients and considering this source of infection to take the necessary precautions when dealing with cases analogous to our case.

## Ethical approval

Ethical approval was taken from Medical Research Center (MRC) Qatar before submission of this manuscript.

## Sources of funding

The publication of this manuscript is supported by Qatar National Library (QNL).

## Author contribution

Mohammed Alnajjar is the corresponding author who identified the case, reviewed literature, and wrote the manuscript. Abdulrahman Al-Mashdali helped in manuscript writing, doing a review for literature. Nedia Nefatti helped in identifying the case, reviewing literature, and doing final review and approval for the manuscript.

## Research registration

N/A.

## Guarantor

Mohammed Alnajjar E-mail: Malnajjar1@hamad.qa.

## Consent

Written informed consent was obtained from the patient for publication of this case report and accompanying images. A copy of the written consent is available for review by the Editor-in-Chief of this journal on request.

## Provenance and peer review

Not commissioned, externally peer-reviewed.

## Declaration of competing interest

All authors involved declared no potential conflicts of interest.
